# The Influence of Learning on Host Plant Preference in a Significant Phytopathogen Vector, *Diaphorina citri*

**DOI:** 10.1371/journal.pone.0149815

**Published:** 2016-03-01

**Authors:** Dara G. Stockton, Xavier Martini, Joseph M. Patt, Lukasz L. Stelinski

**Affiliations:** 1 Department of Entomology and Nematology, University of Florida, Citrus Research and Education Center, Lake Alfred, Florida, United States of America; 2 United States Department of Agriculture, Agricultural Research Services, U.S. Horticultural Research Laboratory, Ft. Pierce, Florida, United States of America; INRA-UPMC, FRANCE

## Abstract

Although specialist herbivorous insects are guided by innate responses to host plant cues, host plant preference may be influenced by experience and is not dictated by instinct alone. The effect of learning on host plant preference was examined in the Asian citrus psyllid, *Diaphorina citri*; vector of the causal agent of citrus greening disease or huanglongbing. We investigated: a) whether development on specific host plant species influenced host plant preference in mature *D*. *citri*; and b) the extent of associative learning in *D*. *citri* in the form of simple and compound conditioning. Learning was measured by cue selection in a 2-choice behavioral assay and compared to naïve controls. Our results showed that learned responses in *D*. *citri* are complex and diverse. The developmental host plant species influenced adult host plant preference, with female psyllids preferring the species on which they were reared. However, such preferences were subject to change with the introduction of an alternative host plant within 24–48 hrs, indicating a large degree of experience-dependent response plasticity. Additionally, learning occurred for multiple sensory modalities where novel olfactory and visual environmental cues were associated with the host plant. However, males and females displayed differing discriminatory abilities. In compound conditioning tasks, males exhibited recognition of a compound stimulus alone while females were capable of learning the individual components. These findings suggest *D*. *citri* are dynamic animals that demonstrate host plant preference based on developmental and adult experience and can learn to recognize olfactory and visual host plant stimuli in ways that may be sex specific. These experience-based associations are likely used by adults to locate and select suitable host plants for feeding and reproduction and may suggest the need for more tailored lures and traps, which reflect region-specific cultivars or predominate Rutaceae in the area being monitored.

## Introduction

Although herbivorous insects rely heavily on innate olfactory and/or visual preferences to locate and select host plants [1–2], innate responses may be shaped by the organism’s experiences in a process known as learning [3–5]. Insects are not only capable of learning, but can accomplish highly complicated tasks as a result. Fruit flies can associate odor, color, and visual patterns with both appetitive and aversive conditions [6–7]; and grasshoppers have the sophisticated ability to learn visual stimuli associated with nutritionally valuable food [8–9]. Hymenopterans display some of the most sophisticated learning abilities investigated thus far. The honeybee is capable of learning and interpreting complex waggle dances to encode information about flower location [10–13]. Meanwhile the bumble bee, *Bombus terrestris*, is known to use spatial learning to efficiently locate and navigate flowers [14–15] and recent work has identified observational learning and even false memories in this species [16–18]. However, beyond the models described above, much less is known about learning in other insect taxa, particularly phytophagous Hemipterans. This is important because Hemipterans represent a diverse and threatening group of agricultural pests, many of which transmit plant disease pathogens, particularly those in the monophyletic suborder Sternorrhyncha, which includes aphids, whiteflies, psyllids and scale insects. Current models predict that within a century, the occurrence and abundance of agricultural disease vectors will spread latitudinally as climate change expands the range of migration, and across geographically isolated regions as human movement continues to perpetuate invasions by non-native species [19–21].

To our knowledge, only a few species in the order Hemiptera have been investigated in the context of learning. Patt & Setamou [22] found evidence of olfactory learning in nymphs of the glassy winged sharpshooter (*Homalodisca vitripennis*). Orientation towards a visual target was reportedly influenced by experience in a species of the minute pirate bug, *Orius majusculus* [23]. Finally, visual and olfactory learning in the context of host preference have been documented in the predatory bug, *Rhodnius prolixus* [24–28]. While there is a general lack of knowledge about learning in Hemipterans, even less is known about how learning may affect pest management [29]. Furthermore, the increased demand for pest management strategies, alternative to traditional synthetic pesticides, necessitates investigation of the behavioral and cognitive ecology of agricultural pests.

Of current concern is the introduction and spread of the Asian citrus psyllid, *Diaphorina citri* Kuwayama (Hemiptera: Liviidae), a phloem-feeding citrus pest that transmits *Candidatus* Liberibacter asiaticus (*C*Las), the putative causal agent of citrus greening disease or huanglongbing (HLB) [30–33]. Management of HLB relies on the intensive use of insecticides to reduce *D*. *citri* populations [31]; however, insecticide resistance has recently been detected in *D*. *citri* [34]. The dependence upon chemical controls in the face of emerging insecticide resistance, along with the recent concerns about neonicotinoids, necessitates the development of alternative control techniques for managing *D*. *citri* populations [35–36]. As a result, better tools are needed to detect and monitor this pathogen vector. Development of successful alternative control techniques, such as trap cropping and ‘attract and kill’ strategies will require a thorough understanding of the insect’s ecology and cognitive abilities with respect to finding host plants and conspecifics [37].

Currently, we know that *D*. *citri* employ several sensory modalities when selecting hosts including vision [38–41] gustation [41], audition (substrate-borne) [42–43], and olfaction [41, 44–46]. Furthermore, the response of the insect to olfactory cues is affected by bacterial infection of plants, which changes the bouquet of volatiles released from infected plants [47–48]. To some extent, the behaviors of *D*. *citri* have already been exploited for pest management. Traps for monitoring *D*. *citri* populations have been optimized to exploit color preferences [40] and olfactory lures based on preferred host plant volatile profiles are under development [49–50]. However, the success of such applications has been limited, and it is unclear whether the preferences displayed by *D*. *citri* are learned or innate.

Previous research on feeding behavior in adult *D*. *citri* indicates a possible role for experience in host selection and acceptance [51], despite strong innate responses to certain host plant volatiles [38, 41, 44, 46]. It remains unclear whether information about stimuli, perceived by developing psyllids, is retained and used in host plant selection as reproductively mature adults. This is an important pest management consideration because if *D*. *citri* host preferences are experience-dependent, then differences in local citrus cultivar abundance may influence monitoring (i.e., orientation to a target) and the development of alternative control measures (i.e., selection of trap crop cultivar) [51].

The goals of the present study were to investigate the extent of associative learning in *D*. *citri* in the form of simple and compound conditioning tasks while exploring memory duration, and the relative salience of visual versus olfactory information. Due to the economic importance of this pest species, an ancillary goal was to determine the factors that may contribute to effective application of behavioral modification as a future management tool for *D*. *citri* and, by extension, other phytopathogen vectors. For this reason, we sought to study learning in the context of host preference. If learning plays an important role in *D*. *citri* host plant preference, traps and lures tailored to regional *Citrus* or ornamental *Murraya* diversity and abundance may enhance the efficacy of current monitoring and disruption techniques.

## Materials and Methods

### Insect colony and Host Plant Maintenance

*D*. *citri* were obtained from a *C*Las-free colony maintained at the University of Florida Citrus Research and Education Center in Lake Alfred, Florida. The psyllids were originally obtained from Valencia orange (*Citrus sinensis* L.) trees and were subsequently maintained on Valencia orange and orange jasmine (*Murraya paniculata* (L.) Jack.). The trees were fertilized with a granular fertilizer monthly, and weekly with a soil drench fertilizer (MaxiGro^™^). To establish colonies reared on a specific host plant species, the psyllids from the general colony were moved into cages with either potted orange jasmine or sour orange (*Citrus* x *aurantium* L.) plants. After initial oviposition, general colony adults were removed to ensure that all developing insects were only experienced with the developmental host plant species; either orange jasmine or sour orange. All colonies were maintained at 28°C under a L14:D10 light cycle. To maintain *D*. *citri* reproduction and ensure maximum plant health, host plants were rotated out of colony cages once per month.

### Y-tube Behavioral Assay Set-up

Olfactory and visual preference tests were performed with a Y-tube apparatus with a 1 cm inner diameter. The length of the Y-tube measured 13 cm from the crux to the release end. The two arms of the Y-tube measured 8 cm from the crux to the odor source ends. The arms of the Y-tube received charcoal filtered and humidified air pumped at 0.2 liters per minute (LPM) from a flowmeter (ARS Inc., Gainesville, FL). The Y-tube was mounted vertically with two white compact fluorescent lights (Sylvania; 13W, 800 lumens) suspended at equal heights from the distal ends of the Y-tube arms. A vertical Y-tube mount was used due to positive phototaxis and negative geotaxis displayed by *D*. *citri* [52–53]. White bulbs were used unless otherwise stated. In visual conditioning experiments involving different light colors, the white bulbs were replaced with colored compact florescent bulbs of the same intensity (Mood-lites^®^; 13W, 800 lumens).

To remove the effects of possible differences in heat and light intensity between the different bulbs, the bulbs were presented within a ‘shade’ container that was constructed from white cardstock (white bulb: 81°C unshaded, 39°C shaded; blue bulb: 80°C unshaded, 38°C shaded). This diffused the light and created a consistent 5 cm buffer between the bulbs and the arms of the Y-tube. Using a shade, the heat measured on the arms of the Y-tube was approximately 26°C regardless of bulb color. Ambient room temperature was approximately 23°C. Temperature was measured with a Fluke 62 Max+ Infrared (IR) thermometer. To control for positional bias, the Y-tube was rotated 180° every 10 trials. To control for potential chemical deposition, which could influence *D*. *citri* behavior [45], the Y-tube was cleaned and replaced every 5 trials. All glassware was cleaned with Sparkleen detergent (Fischerbrand), rinsed with distilled water, rinsed again with acetone, and dried in an oven at 80°C for at least 15 min. Each trial lasted 300s, or until an individual made a selection, which was designated as a minimum 1 cm entry into a particular arm. When a selection was made, the insect was removed from the Y-tube and the trial was ended. Individuals that failed to make a selection within 300 s were designated “nonresponsive.” Data were collected only on days when the response rate was above 80%. The usual response rate was about 90–95% but varied based on barometric pressure. The latency to selection and orientation of the selected arm data (left or right) were recorded in addition to odor selection data. Time to selection data were used to compare response time between male and female *D*. *citri*, as well as, naïve and experienced *D*. *citri* in experiment 3.

### Experiment 1: Experience-Dependent Host Plant Preference

To determine the effect of host plant experience on adult female host plant preference, *D*. *citri* were reared as described above on either sour orange (SO) or orange jasmine (OJ) for two generations. Female *D*. *citri* collected from each host plant colony were then assayed using a Y-tube olfactometer for host plant odor preference. Each arm was baited with odor from either 0.25 g sour orange leaves or 0.25 g orange jasmine leaves. Foliage used in testing was obtained from caged, undamaged plants that had never been fed upon. The age of the plants used in the olfactometer assays was the same as the plants used in the colonies; approximately 2 years. Both sets of plants were treated similarly (e.g., watering, pruning and fertilizer schedules) with the exception of *D*. *citri* herbivory. Only young flushing shoots were used and all leaves used in this experiment were visually approximated to maintain consistent leaf age. Adult psyllids used in these tests ranged in age from 4–7 days post-emergence. Only females were used in experiment 1 due low male response level in preliminary tests.

To determine the plasticity of such preferences, shifts in host plant preference after short-term experience feeding on an alternative host plant species was measured in adults. In this test, four-day old adult females reared on orange jasmine were moved to sour orange and assayed for orange jasmine or sour orange preference at 0-, 24-, 48-, and 72 hrs post host plant transfer. To control for handling and transfer of *D*. *citri*, a separate group was moved from the original host plant (orange jasmine) and placed on new orange jasmine plants in a different cage. These psyllids were assayed at the same time points as the experimental group. This experiment was repeated using sour orange as the natal host plant and orange jasmine as the novel adult host plant.

### Experiment 2: Single Stimulus Conditioning

**Fig 1:**
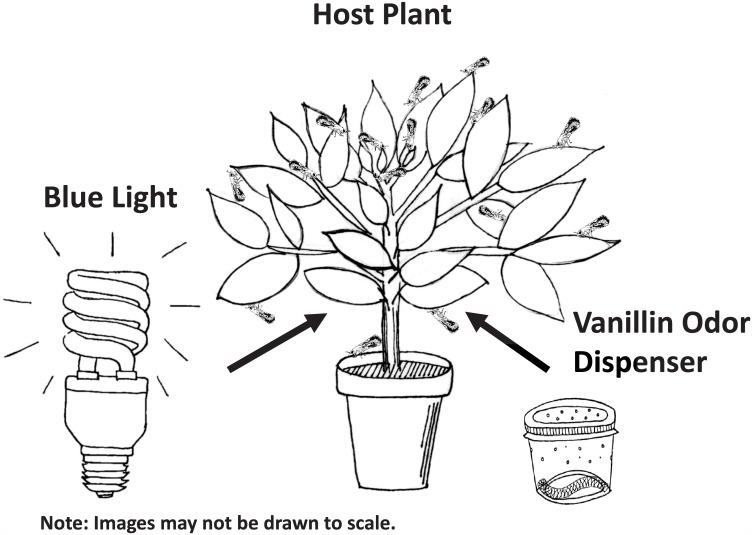
Experimental conditioning procedure. In single stimulus tests, either blue light or vanillin were paired with the host plant. In compound conditioning, all test groups received simultaneous presentation of the visual and olfactory stimuli.

Two experiments were conducted to investigate differences in stimulus acquisition by adult psyllids across sensory modalities. The first experiment evaluated single stimulus conditioning toward a novel, non-host plant associated volatile, vanillin, while the second experiment evaluated single stimulus conditioning to another novel, non-host plant associated stimulus, blue light. In the first experiment, adult *D*. *citri* were released onto caged sour orange trees baited with vanillin (Sigma-Aldrich; CAS 121-33-5). Baits were created by adding 5 ml of a 2.5% ethanolic vanillin solution to a cotton wick. Vanillin was dissolved into solution with 100% ethanol. To prevent direct contact by *D*. *citri* with vanillin, the wicks were enclosed in perforated plastic cups with lids. One bait cup was placed inside the pot of each sour orange plant. *D*. *citri* were allowed to feed freely on the vanillin baited plants for 72 hrs (Fig 1). After 72 hrs, male and female *D*. *citri* were assayed for orientation response to vanillin using the Y-tube olfactometry described above. One arm of the Y-tube was baited with 1ml 2.5% vanillin solution on a cotton wick. The other arm was used as a control and was baited with 1 ml of ethanol on a cotton wick. The wicks were air dried for 30 min prior to use to allow the ethanol to fully evaporate. The results of the vanillin experienced *D*. *citri* were compared with naïve *D*. *citri*.

In the second experiment, adult *D*. *citri* (age non-specific) were released onto caged sour orange trees illuminated with blue light (Mood-lites^®^; compact florescent, 13W) (Fig 1). *D*. *citri* were allowed to feed freely on the blue illuminated plants for 72 hrs. After 72 hrs, male and female *D*. *citri* were assayed for orientation response to blue light using modified Y-tube olfactometry. The test treatment arm of the Y-tube was illuminated with blue light as described above, while the control arm was illuminated with white light. The illumination from each light source was isolated by using a solid white divider that was placed between the two arms of the Y-tube, resting upon the crux.

The results of the blue-light experienced *D*. *citri* were compared with naïve *D*. *citri*. All adult *D*. *citri* were at least 4 days old (fully sclerotized and reproductively mature) before use in any part of experiment 2.

### Experiment 3: Compound Conditioning

To evaluate the relative salience of olfactory versus visual stimuli, as well as, the ability to acquire complex multi-modal information, compound conditioning was performed on male and female *D*. *citri* using the olfactory stimulus, vanillin, and the visual stimulus, blue light. *D*. *citri* (age non-specific) were released onto caged sour orange trees illuminated with blue light and baited with 5 ml of 2.5% vanillin solution. *D*. *citri* were allowed to feed freely on the plants for 72 hrs. After 72 hrs, male and female *D*. *citri* were placed in one of six test groups and assayed using Y-tube olfactometry (Table 1). The test groups represented Y-tube choice-test permutations, which quantified responses to vanillin and blue light independently and as a compound stimulus. The behavioral results of the experienced *D*. *citri* were compared with naïve *D*. *citri*. Similar to experiment 2, all adult *D*. *citri* were at least 4 days old before use in any part of this study.

**Table 1:** Compound conditioning experimental test groups. Each group differs only in stimulus presentation during testing. Each single stimulus is the presentation of the olfactory or visual stimuli alone. The compound stimulus is presentation of the olfactory and visual stimuli simultaneously.

Test Group	Arm 1	Arm 2
a	Olfactory	Blank
b	Visual	Blank
c	Olfactory	Visual
d	Olfactory +Visual	Blank
e	Olfactory +Visual	Olfactory
f	Olfactory +Visual	Visual

### Statistical Analysis

Y-tube data from the host preference tests and the simple and compound conditioning tests were analyzed with Chi-square tests for within-group comparisons and Chi-square 2 x 2 contingency tables for between-group comparisons, alpha ≤ 0.05. Within-group comparisons were performed for selection differences towards either arm of the Y-tube (i.e. orange jasmine odor versus sour orange odor; vanillin odor versus blank; white light versus blue light). Between-group comparisons were performed for overall selection differences between groups (i.e. orange jasmine psyllids versus sour orange psyllids; naïve psyllids versus experienced psyllids). Differences between groups at each time point in Experiment 1 were calculated with chi-square contingency tables. Standard error was calculated for binomial data where *p* is the proportion selecting the target, *q* is the proportion selecting the alternative target, and *n* is the number of Bernoulli trials.

$$SEx=\sqrt{\frac{pq}{n}}$$

Latency data from the compound conditioning experiment were compared using a generalized linear model with a Gaussian distribution. The model was simplified following a stepwise deletion to remove insignificant interactions (α > 0.10). Only latency data from single stimulus versus control tests were included in those analyses. Latency data were not reported for single stimulus conditioning experiments because the sample size was too small to provide adequate statistical power.

At least two replicates, and as many as eight, were performed for each experiment. The number of individuals tested per replicate was dependent on the variation in response and the number of individuals required for statistical power for that particular experiment. For the analyses described above, the data from all replicates within a given experiment were pooled. All analyses were run in R (Version 3.1.3; the R Foundation for statistical software R; Vienna, Austria).

## Results

### Experiment 1: Host Plant Preference

**Fig 2:**
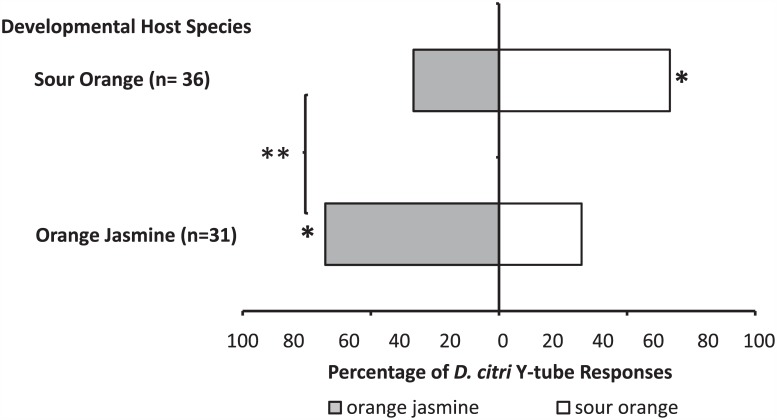
Developmental host plant preference. Differences in female *D*. *citri* preference based on natal host plant species. Asterisks within bars indicate statistically significant differences within groups, while asterisks associated with a bracket indicate differences between groups (χ^2^ test, *: < 0.05, **: < 0.01).

Between group comparisons of the overall response of sour orange-experienced psyllids and orange jasmine-experienced psyllids suggests that host plant preference was significantly affected by natal host plant type (χ^2^ = 7.89, df = 1, p = 0.005) (Fig 2). Within group comparisons showed that female *D*. *citri* reared on sour orange plants preferred sour orange to orange jasmine leaf volatiles (χ^2^ = 4, df = 1, p = 0.045). Conversely, female psyllids reared on orange jasmine significantly preferred orange jasmine odor as compared with sour orange (χ^2^ = 3.9, df = 1, p = 0.048).

**Fig 3:**
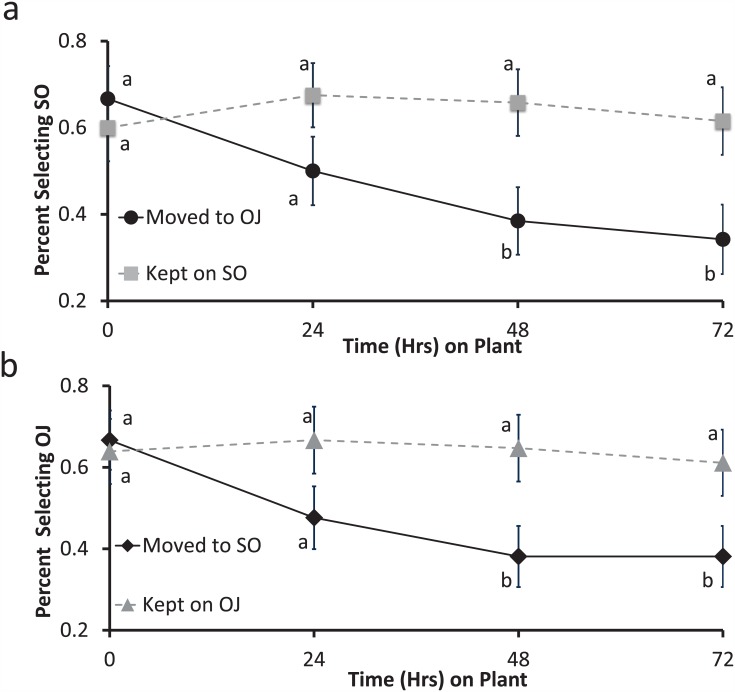
Experience dependent host plant preference. Shifts in preference after short-term adult experience feeding on the alternative host plant species. The labels “Kept on SO” (sour orange) or “Kept on OJ” (orange jasmine) represent the control groups—*D*. *citri* maintained on a single host plant species for the duration of the study. The experimental groups are labeled as “Moved to SO” or “Moved to OJ.” Significant differences between groups at each time point are indicated by different letters (χ^2^ test, α≤0.05).

However, when insects reared on sour orange were transferred to orange jasmine plants as adults, host plant preference shifted towards orange jasmine 48 hrs post-transfer (Fig 3a) (χ^2^ = 5.76, df = 1, p = 0.016). Similarly, a transfer of adult female *D*. *citri* from the developmental host plant species, orange jasmine, to the alternative host plant species, sour orange, changed those preferences in favor of the most recent host plant that was experienced (Fig 3b). By 48 hrs post host plant transfer, *D*. *citri* significantly preferred sour orange compared with *D*. *citri* that had constant exposure to the developmental host plant, which maintained preference for orange jasmine (χ^2^ = 5.32, df = 1, p = 0.021).

### Experiment 2: Single Stimulus Conditioning

**Fig 4:**
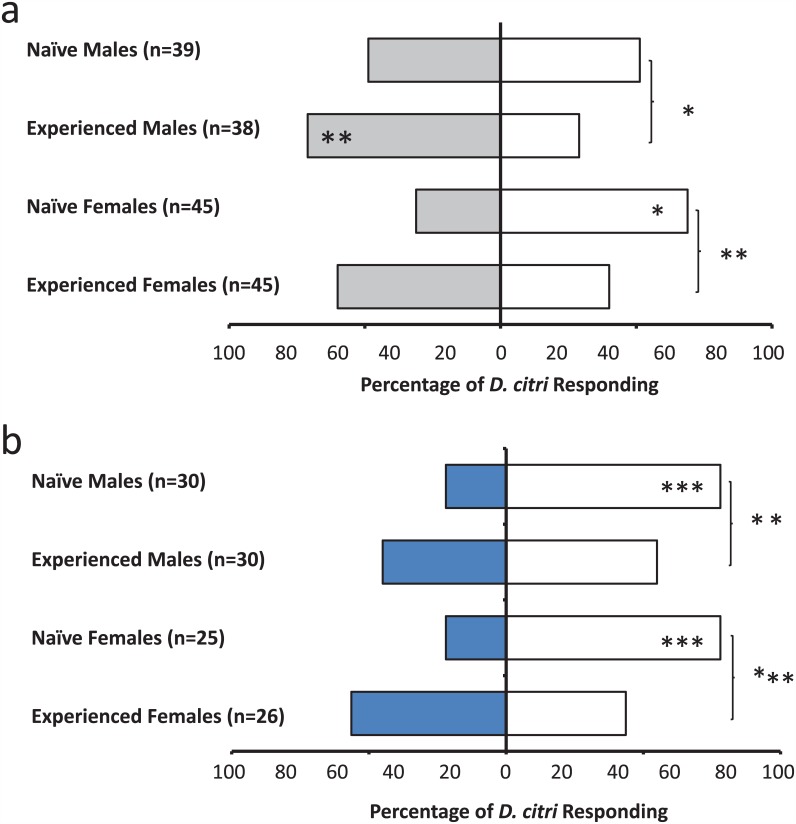
Learned response to a novel olfactory stimulus (a) and a visual stimulus (b). (a) Light gray bars indicate responses to a novel olfactory stimulus (vanillin odor). (b) Blue bars indicate the responses to a novel visual stimulus (blue light). (a, b) White bars indicate responses to the blank control. Asterisks (*) within bars indicate statistically significant differences within groups selecting arm A or arm B, while asterisks (*) outside bars indicate differences between naïve and experienced insects (χ^2^ test, *: < 0.05, **: < 0.01, ***: <0.001).

There was a significant effect of treatment in the single stimulus learning experiments, with experienced insects showing greater response to the test stimulus than naïve insects. Feeding experience on vanillin-baited plants for 72 hrs was sufficient to produce a significant change in response to that volatile in adult *D*. *citri* (Fig 4a). Compared to naïve insects, experienced females (χ^2^ = 7.57, df = 1, p = 0.006) and males (χ^2^ = 3.99, df = 1, p = 0.045) showed significantly greater selection of vanillin. Similarly, feeding experience on plants illuminated with blue light induced a significant change in response compared with naïve insects in both females (χ^2^ = 9.99, df = 1, p = 0.002) and males (χ^2^ = 4.84, df = 1, p = 0.027) (Fig 4b).

### Experiment 3: Compound Conditioning

**Fig 5:**
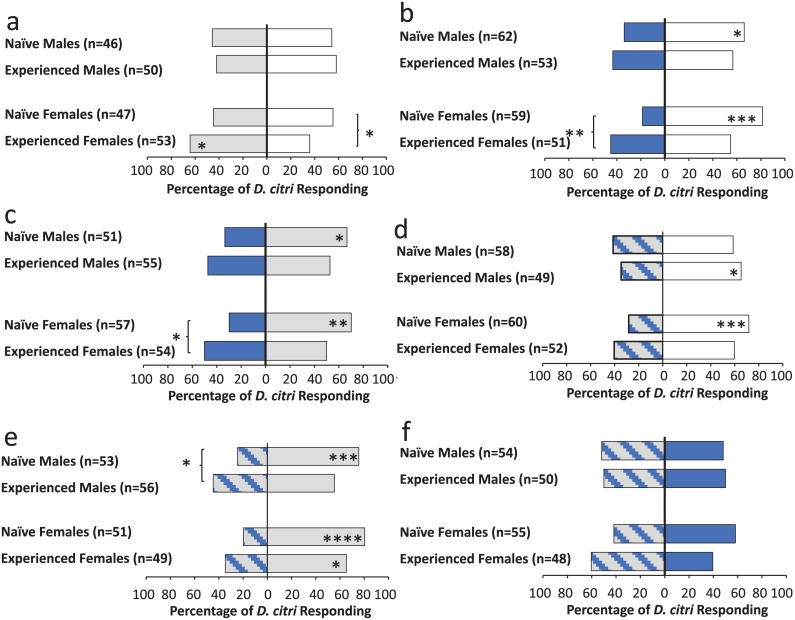
*D*. *citri* responses after compound conditioning to six test conditions (a-f). Gray bars represent the olfactory stimulus (vanillin odor), blue bars represent the visual stimulus (blue light), striped blue and gray bars represent the compound stimulus (vanillin odor + blue light), and white bars indicated a blank control. Asterisks (*) within the bars indicate statistically significant differences within a group selecting arm A or arm B (χ^2^ test, *: < 0.05, **: < 0.01, ***: < 0.001), ****: <0.0001. Asterisks (*) outside the bars indicate differences between a set of naïve and experienced insects for a given test condition (χ^2^ test).

Experience with a bimodal compound stimulus (vanilla odor + blue light) resulted in significantly different responses in female *D*. *citri* to each stimulus when presented individually (Fig 5a and 5b). In a naïve state, female *D*. *citri* oriented towards vanillin at the same rate as the unscented control arm, meaning that response to vanillin was neutral. However, after experience, this percentage increased by approximately 19%. Between group comparisons showed that this difference was statistically significant (χ^2^ = 3.81, df = 1, p≤0.05). Naïve female response to blue light increased similarly after experience, from 19 to 45% (χ^2^ = 8.96, df = 1, p = 0.003). Males did not show increases in response to each stimulus when presented individually.

When the two stimuli were presented on either end of the Y-tube simultaneously, preference shifted as a result of experience (Fig 5c). Naïve males (χ^2^ = 5.67, df = 1, p = 0.02) and females (χ^2^ = 9.28, df = 1, p = 0.002) preferred the arm emitting the olfactory stimulus, while insects that had experienced a combination of vanilla odor + blue light selected the colored and scented arms equally.

When the compound stimulus was presented opposite of the blank control, there were no differences between groups (Fig 5d). However, there was a significant difference in naïve female selection, with preference shifted towards the control arm (χ^2^ = 11.27, df = 1, p<0.0008). This difference was abolished after experience—experienced females showed no preference for either the blank arm or the arm with the compound stimulus. The opposite trend occurred for males; naïve males showed no preference between the compound stimulus and the control; whereas experienced males preferred the control (χ^2^ = 4.59, df = 1, p = 0.03).

The final two tests involved presenting the compound stimulus opposite each of the stimuli individually (Fig 5e and 5f). When the compound stimulus was presented opposite of the olfactory stimulus alone, experienced males showed an increase in response to the compound stimulus (χ^2^ = 4.85, df = 1, p = 0.03) (Fig 5e). There was no difference in female response, (χ^2^ = 2.88, df = 1, p = 0.09). When the compound stimulus was presented opposite of the visual stimulus alone (Fig 5f), there was no statistically significant difference between naïve and experienced insects, although experienced females showed an 18% increase in response to the compound stimulus (χ^2^ = 3.55, df = 1, p = 0.059).

**Fig 6:**
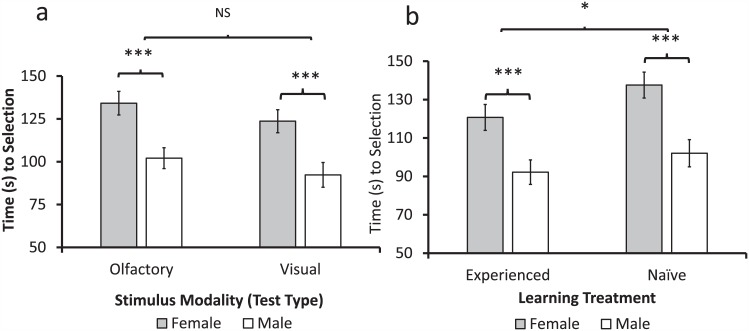
Latency to selection for *D*. *citri*. Differences in selection time in female (gray bars) and male (white bars) psyllids based on (a) the stimulus modality of the test (olfactory or visual test) and (b) the learning treatment applied to the insects (naïve or experienced). Asterisks (*) indicate significant differences *: < 0.05, **: < 0.01, ***: < 0.001).

In addition to behavioral choice data, we also collected latency data. These results revealed differences in response time when making a selection in the 2-choice olfactometer (Fig 6). Comparing the latency to selection for all *D*. *citri* during single stimulus tests using a GLM, there was a significant effect of sex (females compared to males) and treatment (naïve insects compared to experienced insects) on the time needed for selection (Table 2). All of the interactions among sex, treatment, and the sensory modality of the tests (olfactory tasks compared to visual tasks) were insignificant at α < 0.10 and were consequently removed from the model. Time to selection was significantly greater in female *D*. *citri* as compared to males (Fig 6a and 6b). Additionally, experience was associated with a small but significant decrease in response time compared to naïve insects (Fig 6b). No significant differences were observed between the olfactory and visual tasks (Fig 6a).

**Table 2:** Results from the GLM with Gaussian distribution. Results based on the latency data associated with the single stimulus tests in the compound conditioning experiment. All non-significant interactions were removed (α >0.10).

Factor	Df	F	P-value
Sex	1, 402	22.32	<0.0001
Treatment	1, 402	3.94	0.048
Test	1, 402	2.36	0.125

## Discussion

The host plant preference tests in Experiment 1 compared the behavioral response of *D*. *citri* to familiar or novel host plant species. We found that female *D*. *citri* displayed host plant preferences based on experience, such that these insects initially preferred the host plant species on which they were reared. Similar cases of induced host preference have been well documented in *Manduca sexta* [54–55] and *Heliothis zea* [56], where moth larvae reared from egg on a specific host species display feeding preferences for the species on which they were reared. However, the results of a second experiment showed that those initial developmental preferences were subject to change after adult *D*. *citri* were moved to an alternative host plant species. *D*. *citri* preferences shifted to the alternative host plant species within 24–48 hrs following transfer. This suggests that although developmental experience may influence mature adult insect behavior, adult experiences may be more significant in affecting *D*. *citri* host selection, such that the most recently fed upon plant is preferred.

The single stimulus conditioning tests in Experiment 2 investigated the parameters surrounding learning in *D*. *citri* in terms of environment-host plant associations, and showed that male and female *D*. *citri* can associatively learn cues in both olfactory and visual contexts. This reinforces the hypothesis that both olfactory and visual stimuli are important in *D*. *citri* host selection [41] and confirms previous findings of multimodal learning in an artificial feeding assay [51]. Research on multimodal sensory perception in the hawkmoth, *Manduca sexta*, suggests that both forms of sensory information act synergistically, likely aiding in communication between the environment and the foraging insect such that visual cues in combination with olfactory stimuli may provide contextual information [57–59]. In *D*. *citri*, the capacity for visual and olfactory learning may increase the likelihood of detecting suitable hosts when migration and dispersal is required. *D*. *citri* are known to disperse up to 2 kilometers, particularly in search of flushing host plants suitable for oviposition [60–62].

In Experiment 3, we investigated the relative associative strength of olfactory and visual stimuli in terms of biological relevance to *D*. *citri* host plant selection behavior. We predicted that the more salient stimulus would show the greatest rate and proportion of learning [63–64]. The results suggest that female *D*. *citri* learn the individual components of the compound stimulus separately, as well as the compound stimulus as a whole. In addition, while naïve females display strong preference for the olfactory stimulus compared to the visual stimulus, after conditioning, the two stimuli appear to share similar strength of attraction. These findings suggest that female *D*. *citri* actively acquire visual and olfactory information about their host plants, similar to that found in previous work [41, 51]. Interestingly, after the compound presentation of the stimuli, we were unable to demonstrate learning in male *D*. *citri*. Unlike the females, males appeared to show lower capability of learning each stimulus individually and some degree of learned aversion to the compound stimulus. The reasons for this finding are unclear, but it may indicate that the biological significance of the information, or perhaps the way information is stored neurologically, varies depending on the sex of the insect. More experiments are required to better understand these findings and explore those hypotheses.

Along with choice, Experiment 3 also examined the latency prior to behavioral response, which is often used as a measure of decision-making, and in some cases, its own measure of learning [65–66]. We found that experienced *D*. *citri* make target selections faster than naïve insects. The most significant difference was dependent on the sex of the insect, with males making more rapid selections than females regardless of task or experience. This may reflect differences in overall selection strategy between the sexes and may suggest that male psyllids have weaker discriminatory abilities than females, which may help explain the sex differences in learned response rate. This is not surprising considering the reproductive role of females and the importance of oviposition site selection for successful nymph development.

Our investigation was not without some limitations. While our olfactory stimulus, vanillin, was truly novel and is not found in association with the *D*. *citri* host environment, our visual stimulus was not novel. Blue light appears to provoke a strong innate repellent response [38, 39, 50]. In addition, some *D*. *citri* possess a “blue-green” abdominal color-morph and there is increasing evidence that abdominal color is associated with important behavioral differences among psyllids with respect to dispersal and reproduction [60, 67]. However, we avoided strongly attractive colors such as yellow, orange, red, or green [39–40], since previous work has indicated that there is little learned increase in *D*. *citri* behavior when the innate response to the stimulus is positive [51]. Therefore, although the blue light stimulus used in this study does not conform to the characteristics of traditional stimuli used in most learning experiments, we believe it was the appropriate choice for our subject animal. In fact, it revealed a rather interesting result—*D*. *citri* appeared capable of significantly changing their responses, with blue no longer acting as a repellent after conditioning. This suggests that while psyllids may possess intrinsic aversions to certain stimuli, likely evolutionarily selected to help them avoid non-hosts, those aversions are not so hardwired as to be immutable [68–70]. The data shown here suggest that short-term exposure to those stimuli within the context of a reinforcing stimulus, such as food, may override the innate responses of these insects, allowing them to maximally benefit from a complex environment of potential hosts, even those which are on the fringes of their oligophagous range.

Based on these data, we conclude that *D*. *citri* not only learn, but learning may be partially responsible for local host plant preference phenomena [38, 46, 71]. To our knowledge, the findings presented here represent the most extensive study of learning in a sternorrhynchan species. This is significant because sternorrhynchans are an important group of disease vectors and understanding the behavioral ecology involved in host plant preference and selection in these insects is potentially beneficial in the design and implementation of pest monitoring and management programs. Although this work is limited to fairly fundamental questions regarding learning in *D*. *citri* (eg., what types of stimuli are learned, how long memories are retained), and may at this point in time have limited application, we believe that it lays the foundation for further study of learning of this insect and other related sternorrhynchans. It appears likely that *D*. *citri* acquire olfactory and visual information about the host plants with which they have experience. Similar to other psyllid species, visual information appears to be as biologically relevant to *D*. *citri* as olfactory cues in terms of orientation towards a target and association with host plants [72–73]. Given the observed plasticity in response to visual stimuli seen in these experiments, even brief visual experience with a novel, non-traditional, and initially deterrent host may increase the likelihood of future selection for that species, despite repellant visual properties. This could facilitate vector dispersal by providing temporary refuge for psyllids during migration, or even reduce intraspecific competition by introducing novel host plant options; however, further investigation is needed to fully test this hypothesis. For that reason, future *D*. *citri* traps may benefit from increased attention to the visual aspects of design, possibly tailoring color, as well as semiochemical lures to the most prevalent host plant varieties within each citrus growing region.

## Supporting Information

S1 Dataset Original data for Experiments 1–3. Each data file contains those data used for the experiments as described in the text. Each experiment is shown in a separate tab. The latency data collected from experiment 3 is shown in a separate tab as well.
(XLSX)
